# Reliability and Validity of Gaze-Dependent Functional Vision Space: A Novel Metric Quantifying Visual Function in Infantile Nystagmus Syndrome

**DOI:** 10.1167/iovs.17-23229

**Published:** 2018-04

**Authors:** Tawna L. Roberts, Kristi N. Kester, Richard W. Hertle

**Affiliations:** 1 Children's Hospital Vision Center, Akron Children's Hospital, Akron, Ohio, United States; 2 Department of Ophthalmology, Northeastern Ohio Medical University, Rootstown, Ohio, United States

**Keywords:** infantile nystagmus syndrome, gaze dependent visual acuity, visual function, visual acuity

## Abstract

**Purpose:**

This study presents test–retest reliability of optotype visual acuity (OVA) across 60° of horizontal gaze position in patients with infantile nystagmus syndrome (INS). Also, the validity of the metric gaze-dependent functional vision space (GDFVS) is shown in patients with INS.

**Methods:**

In experiment 1, OVA was measured twice in seven horizontal gaze positions from 30° left to right in 10° steps in 20 subjects with INS and 14 without INS. Test–retest reliability was assessed using intraclass correlation coefficient (ICC) in each gaze. OVA area under the curve (AUC) was calculated with horizontal eye position on the *x*-axis, and logMAR visual acuity on the *y*-axis and then converted to GDFVS. In experiment 2, validity of GDFVS was determined over 40° horizontal gaze by applying the 95% limits of agreement from experiment 1 to pre- and post-treatment GDFVS values from 85 patients with INS.

**Results:**

In experiment 1, test–retest reliability for OVA was high (ICC ≥ 0.88) as the difference in test–retest was on average less than 0.1 logMAR in each gaze position. In experiment 2, as a group, INS subjects had a significant increase (*P* < 0.001) in the size of their GDFVS that exceeded the 95% limits of agreement found during test–retest.

**Conclusions:**

OVA is a reliable measure in INS patients across 60° of horizontal gaze position. GDFVS is a valid clinical method to be used to quantify OVA as a function of eye position in INS patients. This method captures the dynamic nature of OVA in INS patients and may be a valuable measure to quantify visual function patients with INS, particularly in quantifying change as part of clinical studies.

Nystagmus is a rhythmic, involuntary oscillation of one or both eyes.^1^ The most common diagnosis is infantile nystagmus syndrome (INS).^1^ INS is a bilateral conjugate eye movement disorder that presents in early infancy and persists throughout life.^1^ The oscillations are clinically uniplanar, but physiologically multiplanar, and have diagnostic increasing velocity slow phases when using eye movement recordings.^2^ Approximately 0.15% of the population has INS.^1,3^ INS may or may not be associated with sensory system deficits (e.g., albinism and achromatopsia).^4–7^ Thus, optotype visual acuity (hereafter visual acuity) is reduced in individuals with INS due to variable combinations of afferent system deficits plus the ongoing eye oscillations.

The clinical and electrophysiologic characteristics of INS in any one patient are time, attention, medication, illness, age, and gaze (eye in orbit) dependent.^1,8^ Eye movement recordings have shown that the waveform and intensity of nystagmus change at different gazes and are often least in an eye position other than primary.^9^ This promotes the adoption of an abnormal head position, allowing individuals' eyes to be positioned at their eccentric gaze null position (i.e., eye position in orbit in which their nystagmus is most dampened). Thus, visual function is variable as a function of gaze in individuals with INS. The treatment goal of the ocular motor oscillation of INS is aimed at improving foveation period dynamics both in and out of the eccentric null zone. Improving the quality of the oscillation has beneficial effects on null zone visual acuity,^10,11^ contrast sensitivity,^12^ visual recognition time, and motion processing. Visual acuity is standard measurement of functional vision and is routinely tested in primary gaze. However, measures of visual acuity in primary position may not be a reliable measure of visual function in patients with INS due its dynamicity (e.g., changes over time, gaze, stress, fatigue, illness, and medication).

Although eye movement recordings are often used to assess visual function (e.g., fixation stability, and eXpanded Nystagmus Acuity Function [NAFX] analyses), they are not presently part of a routine clinical evaluation. Thus, many providers rely on measures of visual acuity to assess visual function in patients with INS. If visual acuity is to be used as an outcome measure for interventional studies in patients with INS, reliability of the method needs to be established. Additionally, the method of collection needs to address its dynamicity (i.e., change in visual function in eccentric gaze positions, over time, with attention, fatigue, illness, and medications). Yang et al. has shown that optotype visual acuity changes in eccentric gaze position.^8^ In typically seeing patients, visual acuity is characterized by best visual acuity. However, in patients with INS, visual acuity may be characterized more specifically by both best and worst visual acuity, gaze position with the best visual acuity (e.g., null position), and the width of the null position.

Hertle et al. introduced the metric gaze-dependent functional vision space (GDFVS) to quantify the dynamic nature of visual acuity (i.e., changes in visual acuity with gaze position) in one metric.^13^ This one metric is able to capture changes that would otherwise be missed if only characterizing changes in best visual acuity. For example, with treatment, one patient with INS may have no change in best or worst visual acuity, but they may have an increase in the width of horizontal gaze (degrees) with best visual acuity, indicating an improvement in visual function. If visual function was only characterized by evaluating best visual acuity, the improvement would be overlooked. However, the metric GDFVS would capture the overall improvement in visual function.

GDFVS is determined graphically. Horizontal eye position is plotted on the *x*-axis, logMAR visual acuity is plotted on the *y*-axis, and the area under the curve (AUC) is calculated. GDFVS is the remaining area not included in the AUC and represents the variation of visual acuity across horizontal visual space as one metric. The better the visual acuity, the smaller the AUC and the larger the GDFVS. Hertle et al.^13^ found a significant increase in GDFVS in patients who underwent treatment for INS. However, although the increase in GDFVS was found to be statistically significant, it is unknown if the increase was clinically significant as test-retest reliability of visual acuity in INS patients across horizontal gaze positions is unknown.

The primary purpose of this study was to assess the reliability of GDFVS by obtaining measures of test–retest of visual acuity and GDFVS in patients with INS and control subjects over varying horizontal gaze positions. A secondary purpose of the study was to show that GDFVS has validity as a measure in a clinical study in patients with INS. GDFVS validity was determined by applying the 95% limits of agreement obtained from the test–retest GDFVS data of experiment 1 to pre- and post-treatment data obtained from INS patients enrolled in a separate study (Hertle et al.).^13^

## Methods

### Study Subjects

The study followed the tenets of the Declaration of Helsinki was approved by the Akron Children's Hospital Institutional Review Board. For both studies, parents provided written parental permission, while subjects <18 years of age provided assent. Subjects 18 years and older provided written informed consent.

In experiment 1, subjects were recruited from Akron Children's Hospital's staff, student, and patient populations to participate in a reliability study of visual acuity across 60° of horizontal gaze positions and to validate the metric, GDFVS. Subjects ≥8 years of age with INS (experimental subjects) and without INS (control subjects) were recruited to participate in the reliability study. Experimental subjects were included if they were diagnosed with INS and control subjects were included if they had visual acuity better than 20/25 in each eye. Subjects (both experimental and control subjects) were excluded from participation if they had a history of acquired brain injury, developmental disabilities that would interfere with the ability to complete the study, or were diagnosed with periodic alternating nystagmus.

In experiment 2, GDFVS data from 85 subjects with oculocutaneous albinism type 1 (OCA1) who had been treated for their INS as part of a separate study^13^ were used to determine whether GDFVS has validity as a measure of the effect of treatment in patients with INS and is referred to here as the INS treatment study. The INS treatment study has been described in detail elsewhere.^13^ In brief, OCA1 subjects with GDFVS data were included in this analysis if they had complete data collected and had follow-up visits for at least 12 months in the INS treatment study. All OCA1 subjects underwent three consecutive treatment modalities following stable visual acuity in spectacle correction: (1) eye muscle surgery, (2) contact lenses, and (3) oral Baclofen. Prior to the aforementioned treatment, spectacle optical correction was given to all patients who met the study's minimum refractive error criteria (myopia ≥ 0.75 diopters [D], astigmatism ≥ 1.50 D, anisometropia ≥ 1.00 D, and fully corrected or symmetrically undercorrected [by up to 1.50 D] hyperopia of ≥+3.50 D [spherical equivalent]) prior to the beginning of the study treatment. Visual acuity was measured using isolated letters surrounded by crowding bars monocularly at 3 meters (M) following the psychometric visual acuity testing protocol established by the Pediatric Eye Disease Investigator Group (HOTV for subjects ≤7 years and Early Treatment Diabetic Retinopathy Study [ETDRS] visual acuity for subjects >7 years). Letters were presented on the M&S SmartSystem (M&S Technologies, Inc., Niles, IL, USA). Subjects were followed every 4 ± 1 weeks until stable visual acuity was achieved (±1 line of previous visual acuity).

### Experimental Setup

In the reliability study of optotypic visual acuity supporting the GDFVS metric, single surrounded visual acuity was measured binocularly over 60° of horizontal gaze from 30° in left gaze to 30° in right gaze in 10° steps following the psychometric visual acuity testing protocol established by the Pediatric Eye Disease Investigator Group electronic ETDRS protocol presented on the Electronic Visual Acuity tester (Emmes Corp., Rockville, MD, USA).^14,15^ All subjects wore their habitual correction during testing. Subjects completed the visual acuity protocol in all seven gazes once and then repeated the visual acuity protocol again in all seven gazes. A time limit to respond was not imposed during testing. Gaze order was randomized to eliminate order bias. The reliability study took approximately 45 minutes to 1 hour to complete, and thus subjects were given breaks as needed. All tests and retests were administered on the same day. Refractive error was not assessed as part of the study, and thus the measured visual acuities are not intended to represent best-corrected visual acuity but rather test–retest reliability.

**Figure 1:**
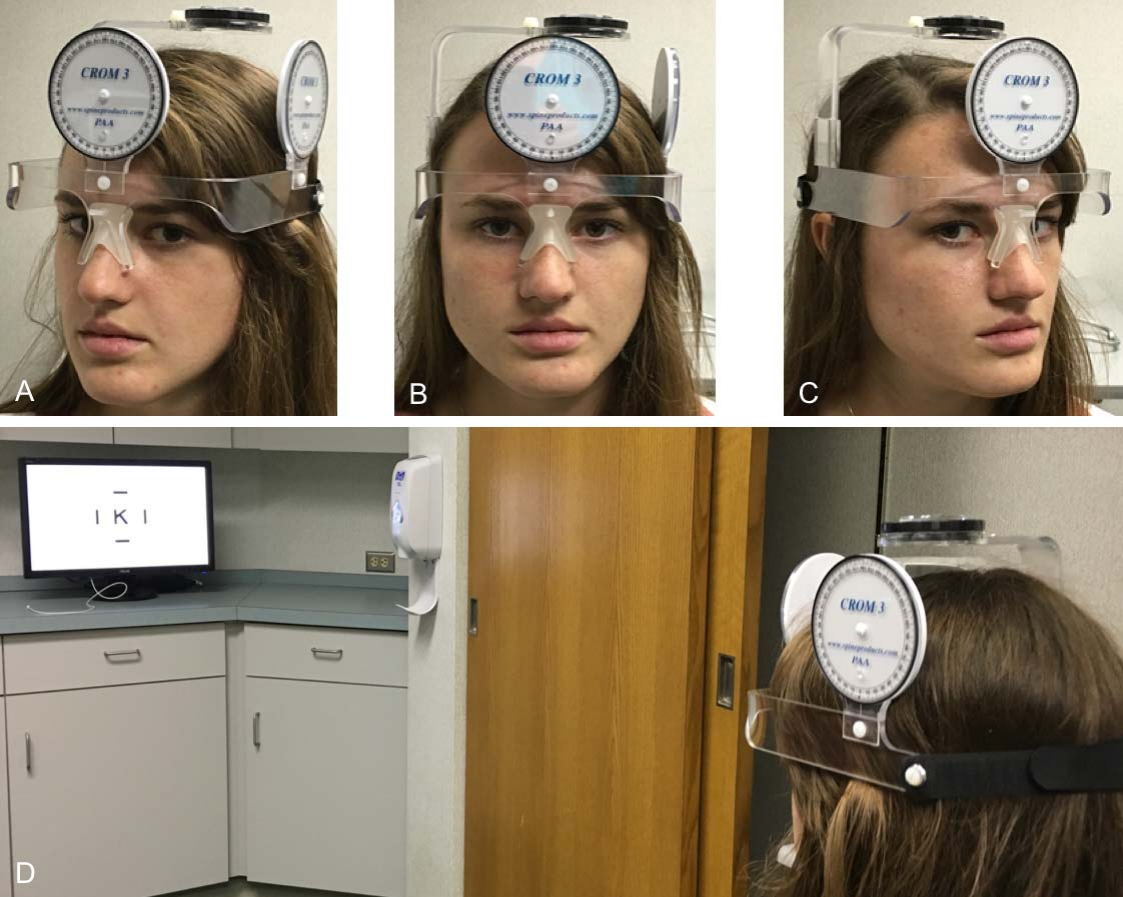
Representative experimental setup with a subject wearing a Cervical Range of Motion device to monitor head position. (A) Subject with a 30° head turn to the right putting the subject's eyes in left gaze (eye in orbit). (B) Subject with a 0° head turn. (C) Subject with a 30° head turn to the left putting the subject's eyes in right gaze (eye in orbit). (D) Subject performing visual acuity while wearing the Cervical Range of Motion device.

Given that patients with nystagmus often adopt an abnormal head position, to ensure subjects maintained proper alignment throughout testing, subjects wore a Cervical Range of Motion 3 device^16^ (Performance Attainment Associates, Lindstrom, MN, USA) as seen in Figure 1. The Cervical Range of Motion device consists of a compass and two ball indicators that allow the head to be secure in the head and neck axial planes. A compass is positioned above the head and measures horizontal head rotation (*z*-axis). One ball indicator is positioned on the forehead to measure lateral head tilt (*x*-axis) and the other ball indicator is located on the left side of the head (above the ear) to measure head extension–flexion (i.e., chin elevation and depression; *y*-axis). Subjects were seated in a stationary chair with their back flat to the chair during testing. The subject's head was placed in primary position and the compass was rotated to the zero position. The subjects' trunk position, compass, and ball indicators were monitored throughout testing to ensure the subjects did not develop trunk or shoulder rotation and that head position was maintained at 0 ± 1° from the intended horizontal head rotation, 0 ± 1° degree of head tilt, and 0 ± 1° head extension–flexion. The experimental setup is shown in Figure 1.

In the INS treatment study reported in Hertle et al.,^13^ gaze-dependent visual acuity was measured binocularly with best correction (spectacles or contact lenses when required) in 40° of horizontal gaze (20° left gaze to 20° right gaze) in 5° intervals using the Pediatric Eye Disease Investigator Group age appropriate protocols (HOTV for subjects ≤7 years and ETDRS^15^ for subjects >7 years) presented on the M&S SmartSystem (M&S Technologies, Inc.). Subjects wore a Cervical Range of Motion 3 device to monitor head position during testing. Visual acuity was obtained in all gazes both before treatment (once visual acuity was stable in spectacles) and after treatment. Subject data and results of the study are found elsewhere.^13^

### Variable Calculations

**Figure 2:**
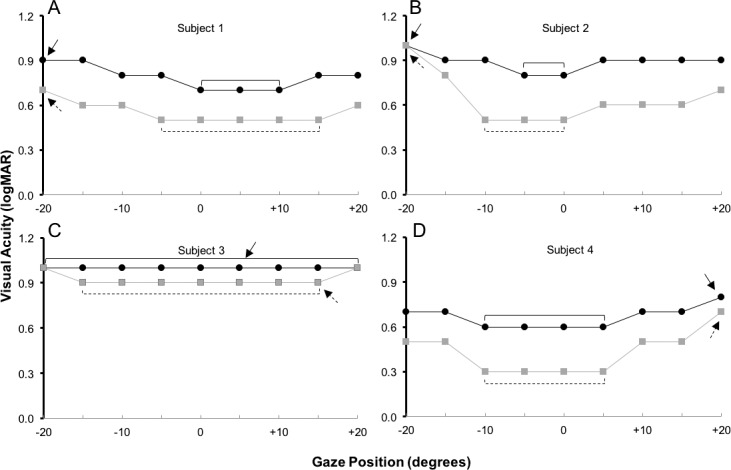
Representation of dynamic changes in gaze-dependent visual acuity (logMAR) across 40° of horizontal gaze (20° right gaze to 20° left gaze) in four subjects who underwent treatment for INS. The black lines represent visual acuity at the pretreatment visit and the gray lines represent visual acuity at the post-treatment visit. For each subject, the solid arrow represents the worst visual acuity and the solid bracket represents the width of horizontal gaze area with best visual acuity at the pretreatment visit. The dotted arrow represents the worst visual acuity and the dotted bracket represents the width of horizontal gaze area with best visual acuity at the post-treatment visit.

For test–retest, visual acuity at each gaze position was transformed from letter score (outcome measure of the electronic visual acuity tester) to logMAR (logMAR = 1.7 − [0.2*letter score]). The smallest line for visual acuity (logMAR) in which three of five presentations were correct was used to represent measures of best visual acuity, worst visual acuity, the width of horizontal gaze (degrees) with best visual acuity, AUC, and GDFVS. Visual acuity was plotted graphically with gaze position on the *x*-axis and visual acuity on the *y*-axis for each individual subject (Fig. 2). The best and worst visual acuity was determined as the best or worst visual acuity measured across the horizontal gaze positions tested (Fig. 2). The width of the horizontal gaze with best visual acuity was determined as the width of horizontal gaze that had consecutive measures of visual acuity equal to the best visual acuity measure obtained over all horizontal gaze positions tested. For example, in Figure 2A, subject 1 had (upper left plot) 0.7 logMAR pretreatment (black line) best visual acuity, 0.9 logMAR worst visual acuity, and the width of horizontal gaze area with best visual acuity was 10° (0 to +10°), whereas the post-treatment (gray line) best visual acuity was 0.5 logMAR, worst visual acuity was 0.7 logMAR, and the width of gaze with best visual acuity was 20° after treatment (−5° to +15°). The change the width of horizontal gaze with best visual acuity was obtained from the 60° of horizontal gaze tested in each subject for test–retest in the reliability study and the 40° of horizontal gaze tested in the INS treatment study.

The AUC was calculated by integration using Matlab (Mathworks, Inc. Natick, MA, USA) with the horizontal eye position plotted on the *x*-axis and logMAR visual acuity plotted on the *y*-axis. In our studies, the ceiling visual acuity was 1.3 logMAR and thus a maximum AUC of 78 logMAR^2^/degrees could be calculated in the test–retest reliability study (60° degrees of horizontal gaze), and 52 logMAR^2^/degrees could be calculated if a subject had 1.3 logMAR visual acuity at each of gaze position tested in the INS treatment study (40° of horizontal gaze). AUC may also be calculated as the product of mean visual acuity obtained across gaze positions multiplied by the width of the gaze range tested. GDFVS for each subject was calculated by subtracting the calculated AUC from the maximum AUC for each study (e.g., 78 logMAR^2^/degrees in the test–retest experiment and 52 logMAR^2^/degrees in the INS treatment study).

### Data Analysis

In the reliability study, the subjects were divided into two groups: group 1, subjects with INS; group 2, subjects without INS and typical visual acuity. Visual acuity test–retest was assessed using intraclass correlation coefficients^17^ in each of the seven gaze positions. Repeated-measures 2-factor ANOVA was used to assess differences in visual acuity test–retest between groups and gaze positions. Bland-Altman plots were generated to further assess differences in GDFVS between visual acuity test–retest in each group.

For subjects who participated in the INS treatment study, all variables were calculated at both the pretreatment visit (i.e., baseline visit with stable visual acuity in spectacles) and the post-treatment visit following the final treatment modality. The data from the INS treatment study were analyzed twice: (1) all original data points, 40° of horizontal gaze in 5° steps and (2) 40° of horizontal gaze in 10° steps. The second analysis allowed the data to be used to determine the validity of the GDFVS metric by using the same step intervals as used in the reliability study. Paired *t*-tests were used to compare the change in outcome variables best and worst visual acuity, width of horizontal gaze with best visual acuity, and GDFVS between the pre- and post-treatment evaluations. Linear regression analysis was used to determine whether there was a relationship between age and the outcome variables. All subjects were included in all analyses regarding best and worst visual acuity and GDFVS. For all analyses concerning the width of horizontal gaze with best visual acuity, subjects were only included if they had at least one gaze position where visual acuity was at least 0.1 logMAR better than the other gazes at the baseline visit. Otherwise, if the width of horizontal gaze area with best visual acuity was 40° at baseline (i.e., equal visual acuity in all gazes) and the subject had an improvement in visual acuity in at least one but less than nine gaze positions, it would falsely appear that the subject had a decrease in the width of horizontal gaze with best visual acuity.

To assess the validity of the GDFVS, the minimum change considered to be a true treatment effect for the subjects in the INS treatment study, and not just variability in the measure, was the 95% limits of agreement (Equation 1) and was computed as the difference of GDFVS of the test–retest subjects.

\newcommand{\bialpha}{\boldsymbol{\alpha}}\newcommand{\bibeta}{\boldsymbol{\beta}}\newcommand{\bigamma}{\boldsymbol{\gamma}}\newcommand{\bidelta}{\boldsymbol{\delta}}\newcommand{\bivarepsilon}{\boldsymbol{\varepsilon}}\newcommand{\bizeta}{\boldsymbol{\zeta}}\newcommand{\bieta}{\boldsymbol{\eta}}\newcommand{\bitheta}{\boldsymbol{\theta}}\newcommand{\biiota}{\boldsymbol{\iota}}\newcommand{\bikappa}{\boldsymbol{\kappa}}\newcommand{\bilambda}{\boldsymbol{\lambda}}\newcommand{\bimu}{\boldsymbol{\mu}}\newcommand{\binu}{\boldsymbol{\nu}}\newcommand{\bixi}{\boldsymbol{\xi}}\newcommand{\biomicron}{\boldsymbol{\micron}}\newcommand{\bipi}{\boldsymbol{\pi}}\newcommand{\birho}{\boldsymbol{\rho}}\newcommand{\bisigma}{\boldsymbol{\sigma}}\newcommand{\bitau}{\boldsymbol{\tau}}\newcommand{\biupsilon}{\boldsymbol{\upsilon}}\newcommand{\biphi}{\boldsymbol{\phi}}\newcommand{\bichi}{\boldsymbol{\chi}}\newcommand{\bipsi}{\boldsymbol{\psi}}\newcommand{\biomega}{\boldsymbol{\omega}}\begin{equation}\tag{1}95\% \ {\rm{limits\ of\ agreement}} = x\ \pm\ 1.96 \times SD,\end{equation}

where *x* is the sample mean and *SD* is the standard deviation of the difference in GDFVS in test–retest.

## Results

### Test–Retest Reliability of Visual Acuity in INS and Control Subjects

**Figure 3:**
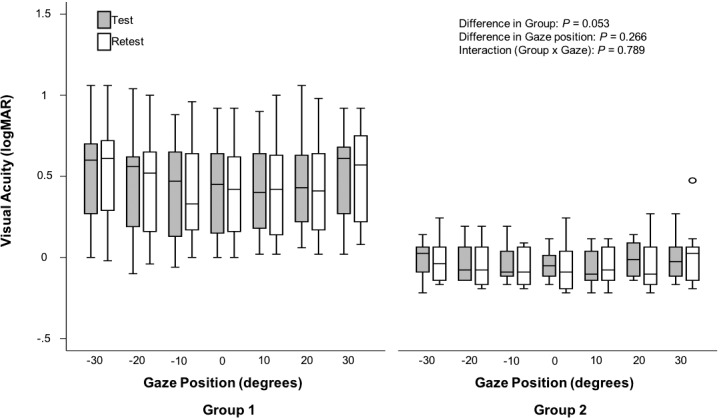
Box plot diagram showing visual acuity (logMAR) in each measured gaze ranging −30° right (eye in orbit) to 30° left (eye in orbit) in 10° steps for group 1 (subjects with INS) and group 2 (subjects without INS and typical visual acuity). Results from the repeated-measures 2-factor ANOVA (group and gaze) are listed in the plot.

Subjects in group 1 (*n* = 20) had a median age of 15.6 years (interquartile range, 12.3, 15.5; range, 8.9 to 47.4) years and subjects in group 2 (*n* = 14) had a median age of 42.1 years (interquartile range, 33.7, 48.4; range, 21.6 to 65.0). Summary characteristics of best and worst visual acuity, width of horizontal gaze with best visual acuity, and GDFVS over 60° horizontal gaze in 10° steps test–retest are found in Table 1. The mean difference in test–retest visual acuity is found in Table 2. On average, subjects in both group 1 and group 2 had a mean difference in visual acuity of less than 0.1 logMAR at each gaze position. The intraclass correlation coefficients were high in both group 1 (≥0.97) and group 2 (≥0.88) for all gaze positions (Table 2). As seen in Figure 3, there were no significant differences in visual acuity test–retest detected between groups (*P* = 0.053) or gaze positions (*P* = 0.266). There was also no evidence of an association between the differences in test–retest visual acuity and gaze position (*P* = 0.79). Additionally, a significant association was not detected between age and the difference in GDFVS (95% CI: −0.07 to 0.03; *P* = 0.492).

**Table 1:**
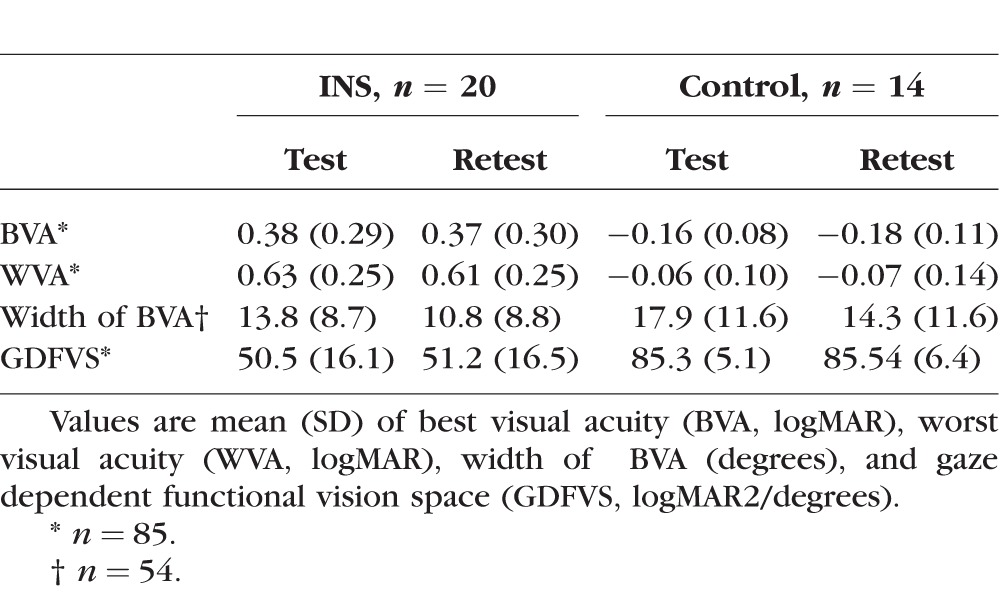
Summary Characteristics for Test-Retest in Experiment 1

**Table 2:**
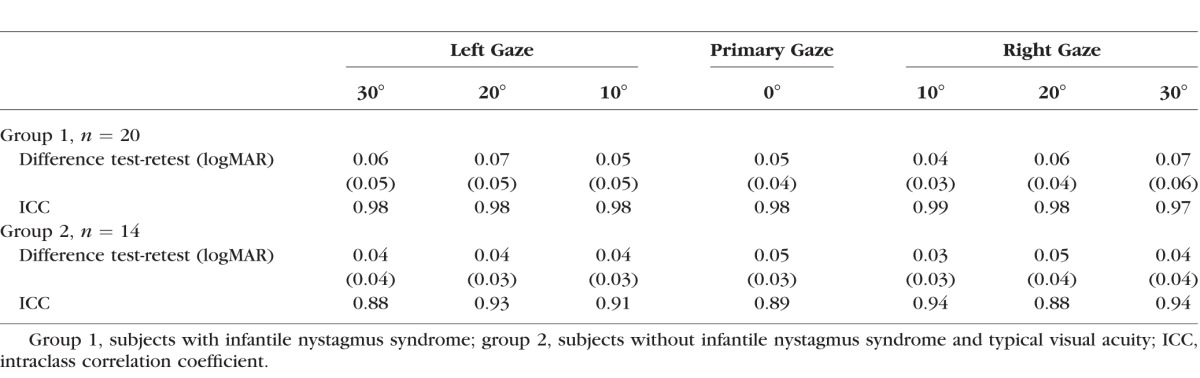
Experiment 1: Mean Differences (Letter Score) and Intraclass Correlation Coefficient From Visual Acuity Test-Retest

**Figure 4:**
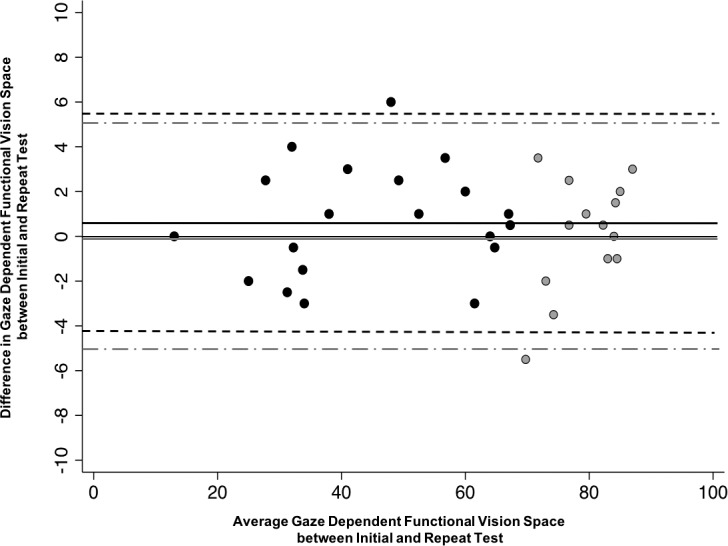
Bland-Altman plot of test–retest difference versus mean GDFVS. A positive difference indicates the repeat GDFVS was better than the initial GDFVS. The black circles represent group 1 (n = 20; subjects with INS) and gray circles represent group 2 (n = 14; subjects without INS and typical visual acuity). Black solid line represents the mean difference in test–retest for group 1 (0.7), black dotted lines represent the 95% limits of agreement for the mean difference for group 1 (−4.2 to 5.6), gray solid line represents the mean difference in test–retest for group 2 (0.1), and gray dotted lines represent the 95% limits of agreement for the mean difference for group 2 (−5.0 to 5.2).

On average, the absolute mean difference in GDFVS for the 60° of horizontal gaze evaluated between test–retest in group 1 was 2.0 ± 1.56 logMAR^2^/degrees and 1.96 ± 1.51 logMAR^2^/degrees for group 2. To apply the test–retest GDFVS results of experiment 1 to the subjects in the INS treatment study, the mean difference in the test–retest group was recalculated using only 40° of horizontal gaze, similar to that measured in the subjects in the INS treatment study to determine the appropriate 95% limits of agreement. The Bland-Altman plot is shown in Figure 4. On average, the absolute mean difference in GDFVS for the 40° of horizontal gaze tested between test and retest was 1.53 ± 1.11 logMAR^2^/degrees for group 1 and 1.50 ± 1.06 logMAR^2^/degrees for group 2. The 95% limits of agreement in subjects with INS was [−4.14 to 3.08], and the 95% limits of agreement for the control subjects without INS and typical vision was [−4.38 to 2.98].

### Change in Visual Acuity and GDFVS in Treated INS Subjects

Previously reported data^13^ from 85 (37% female) patients aged 3.1 to 59.2 years (median age = 11 years; interquartile range, 6.5, 26) with OCA1 and INS were used for this portion of the study. The mean range of follow-up after the initial treatment ranged from 12 to 50 months (mean = 15.1 months). Summary characteristics of best and worst visual acuity, width of horizontal gaze with best visual acuity, and GDFVS over 40° horizontal gaze in 5° steps pre- and post-treatment are shown in Table 3 (note: 54 subjects were included in the width of horizontal gaze with best visual acuity calculations).

**Table 3:**
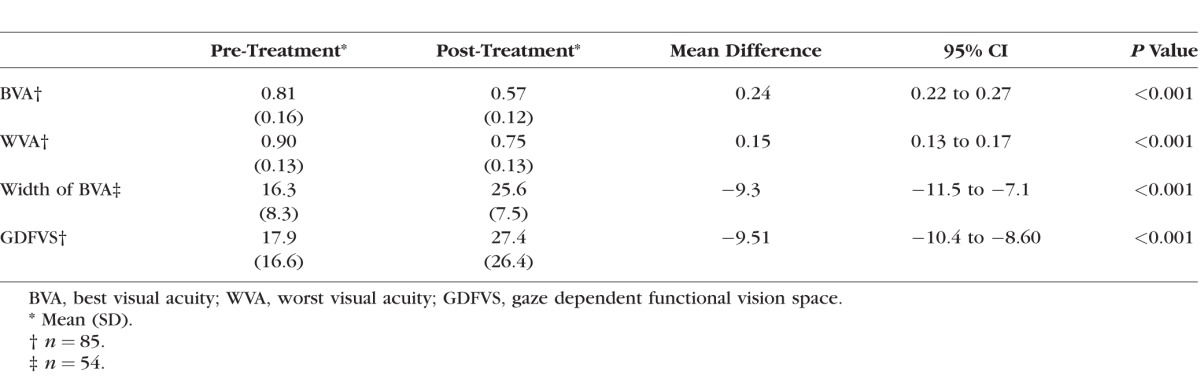
Descriptive and Summary Statistics for the 85 Subjects in the Infantile Nystagmus Treatment Study Pre- and Post-Treatment

**Figure 5:**
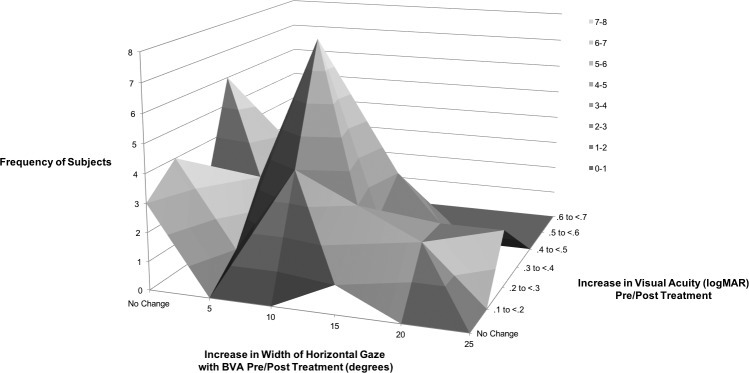
Three-dimensional plot representing the frequency of improvements in visual acuity (logMAR) and increases in the width of horizontal gaze with best visual acuity in 85 subjects treated for INS.

As shown in Table 3, on average, subjects had a significant improvement (*P* < 0.001) between pre- and post-treatment for best visual acuity, worst visual acuity, and a significant expansion of the width of horizontal gaze area with best visual acuity. The AUC also decreased between pre- and post-treatment resulting in a significant increase in GDFVS (*P* < 0.001). Table 4 and Figure 5 show numerical and graphical depictions of the frequency distributions of both the improvement in best visual acuity and the width of horizontal gaze with best visual acuity between pre- and post-treatment. An improvement of at least two lines of improvement in best visual acuity was found in 48% (*n* = 41) of subjects, and 41% (*n* = 35) of subjects had an improvement of ≥10° width of horizontal gaze with best visual acuity. An improvement of both ≥0.2 logMAR best visual acuity and ≥10° width of horizontal gaze area with best visual acuity was seen in 29% (*n* = 25) of subjects. The most frequent combination of improvement (increase in best visual acuity and increase in the width of horizontal gaze with best visual acuity) in subjects (*n* = 8) was represented by an increase in 0.2 logMAR of best visual acuity and an increase in 10° in width of horizontal gaze with best visual acuity.

**Table 4:**
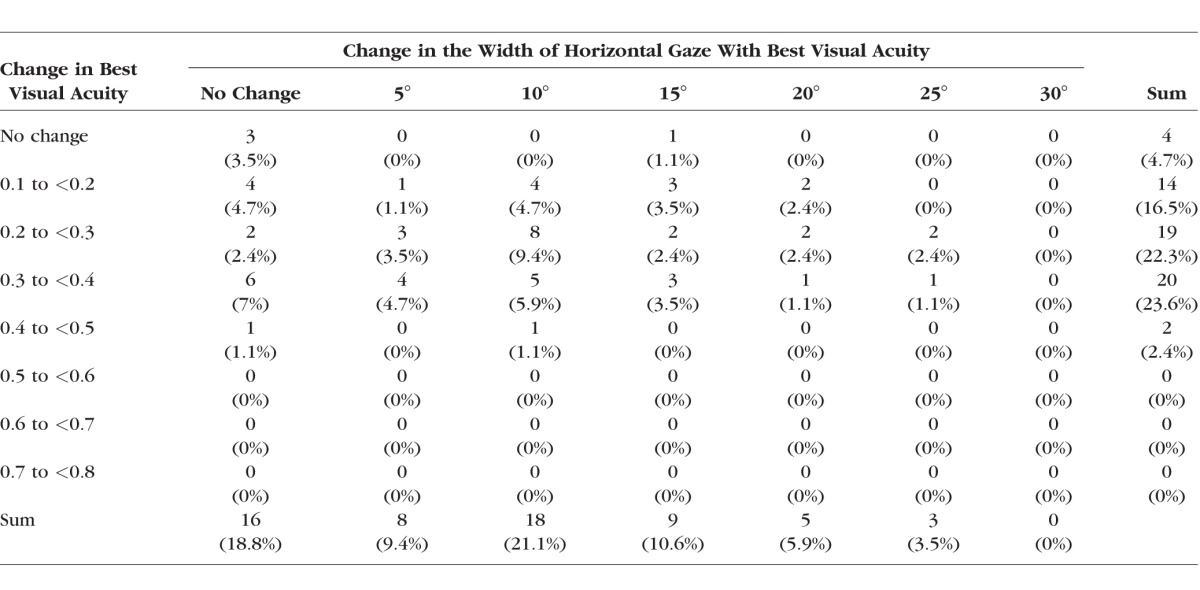
Cross-Correlation Tabulation Representing the Frequency of Subjects Who Had an Improvement in Best Visual Acuity (logMAR) and an Increase in the Width of Horizontal Gaze (Degrees) With Best Visual Acuity Between Pre- and Post-Treatment

Linear regression analysis was performed to determine whether there was an effect of age on any outcome variables. Regression analyses did not detect a significant relationship between age and the change in best visual acuity (95% CI:, −0.003 to 0.001; *P* = 0.438), worst visual acuity (95% CI: −0.001 to 0.002; *P* = 0.541), width of horizontal gaze area with best visual acuity (95% CI: −0.04 to 0.28; *P* = 0.139), or GDFVS (95% CI: −0.76 to 0.70; *P* = 0.938).

Because the reliability study and the INS treatment study had been conducted using different horizontal gaze positions (reliability study: 60° in 10° steps; INS treatment study: 40° in 5° steps), changes in GDFVS in the INS treatment study subjects were calculated using both 5° and 10° steps over the 40° range measured. Both step sizes were shown to have greater changes in GDFVS between pre- and post-treatment (mean change 5° steps: 9.5 ± 4.2; mean change 10° steps: 9.3 ± 4.3) than would be expected from test–retest variability (95% limits of agreement in INS subjects in the reliability study using 10° steps: −4.14 to 3.08). Next, to determine whether there were a difference in outcome whether 5° or 10° steps were used, the pre- and post-treatment GDFVS were compared. A significant difference was not detected when comparing pretreatment GDFVS calculated using 5° steps to pretreatment to GDFVS calculated using 10° steps (mean difference = 0.03; 95% CI: −0.03 to 0.1; *P* = 0.33). However, a significant difference was found when comparing post-treatment GDFVS calculated using 5° steps to post-treatment GDFVS using 10° steps (mean difference = 0.2; 95% CI: 0.11 to 0.35; *P* < 0.001).

## Discussion

Using GDFVS in the evaluation of patients with INS has many advantages: it adds no expense, is a routine clinical test, and testing in multiple gazes provides a more robust measure of visual function. This additional testing requires little extra time and can be used in the absence of eye movement recordings. Additionally, a determination of an area of best visual function may provide patients, clinicians, and vision researchers with a more valuable measure of dynamic visual function.

The difference in GDFVS for test–retest was evenly distributed around zero of the *y*-axis over the entire range of measured GDFVS, suggesting there was no systematic increase or decrease in reliability as a function of visual acuity and subsequently calculated GDFVS (Fig. 4). We also did not find any age effect. Thus, neither visual acuity nor age impacts the validity and reliability of our results over the visual acuity and age ranges tested (e.g., visual acuity of −0.2 to 1.3 logMAR in individuals 8 to 65 years of age).

The reliability of GDFVS determination is dependent on test–retest reliability of visual acuity testing at each gaze used to calculate the AUC and subsequent GDFVS. Our measures of repeatability of visual acuity determination are similar to those reported in typically seeing children,^18^ adults,^14,15,19,20^ and children with amblyopia.^18^ The INS subjects had, on average, 0.05 logMAR difference between test and retest, which was similar to the subjects without INS (0.04 logMAR; Table 2). Intraclass correlation coefficients also show excellent repeatability for visual acuity test–retest in both the subjects with INS and control subjects with normal visual acuity (Table 2). These results support our hypothesis that visual acuity testing as a function of gaze is a valid measure of visual function in individuals with INS.

When applying the GDFVS metric to data from subjects who participated in a previous treatment study for INS, we found a significant increase in GDFVS between pre- and post-treatment, on average, of ∼10 logMAR^2^/degrees, which is nearly twice than would be expected if the difference was due to test–retest variability. The results were similar whether using 5° or 10° step sizes. These data suggest that GDFVS is a valid metric that may be used to quantify changes in best and worst visual acuity. Also, although the mean difference between post-treatment GDFVS and their respective 95% confidence intervals were significantly different, the difference is unlikely to be clinically meaningful. Thus, 10° step sizes may be used instead of 5° steps, making it a more efficient clinical measure. It is also notable that the subjects in the repeatability study were ≥8 years of age, but several subjects (*n* = 24) were younger than 8 years (and performed HOTV rather than ETDRS visual acuity) in the INS treatment study. Subjects younger than 8 years were analyzed separately (data not shown) and were found to have a very similar increase in GDFVS before and after treatment as the subjects ≥8 years (<8 years: 8.88 ± 0.83; ≥8 years: 9.53 ± 0.56), suggesting that the younger children have similar increases in GDFVS as older children and adults.

### Study Limitations

Our study is not without limitations. A limitation of the reliability study is that the experimental group and control group are not age matched. If age were a confounding variable, it would be predicted that the younger subjects in the experimental group would perform worse than the older subjects in the control group.^21^ However, we found no difference in repeatability between the two groups, suggesting that repeatability of visual acuity is adult-like by 8 years of age (youngest in the experimental group). Other investigations have also found similar repeatability in both children >7 years of age^18^ and adults.^14,15,19,20^ Additionally, GDFVS has only been characterized across horizontal gaze in this study, and patients with INS have variable visual acuity in vertical and oblique gaze as well. Thus, this method will only capture visual function in one of three dimensions. Another limitation of our study is that gaze-dependent visual acuity was only measured at the baseline visit and the final outcome visit of the subjects from the INS treatment study. Thus, we are unable to determine which of the three treatment modalities had the largest impact on each individual subject or on the group as a whole. However, the purpose of including those subjects in our analyses was not to characterize the GDFVS as function of treatment type but rather to test the hypothesis that a single metric can be used to capture the dynamic changes that may occur in visual acuity measures in individuals with INS with treatment in the absence of eye movement recordings. Another potential limitation regarding the methodology is that vision may be obscured in one eye at the 30° left and right gaze positions while wearing the Cervical Range of Motion 3 device as shown in Figure 1. Although we did not find a statistically significant difference in repeatability in those gazes compared with the other gazes, suggesting that this did not impact the results of the study, we suggest being mindful of the potential for the Cervical Range of Motion 3 device to obscure one eye when using the device clinically. Last, it is important to note that viewing a stimulus with eccentric head positions while the stimulus is stationary is different than keeping the head still and moving the stimulus to eccentric gazes. Eccentric head position may produce different gaze instabilities with in patients with specific central pathologies. This should be considered when deciding which patients this method should be performed on clinically.

## Conclusions

Our study demonstrates that visual acuity is a reliable measure of visual function in patients with INS when obtained across 60° of horizontal gaze positions. Our results also suggest that the metric, GDFVS, is a reliable clinical method that may be used to quantify visual acuity as a function of eye in orbit. Thus, this method captures one aspect of visual function dynamicity in patients with INS. This measure may be a valuable tool to quantify visual function patients with INS in the absence of eye movement recordings. Additionally, this method is particularly useful in quantifying change in visual function after treatment as part of a clinical study in patients with INS.
